# Antimicrobial Efficacy of Mineral Trioxide Aggregate with and without Silver Nanoparticles

**Published:** 2013-10-07

**Authors:** Mohammad Samiei, Mohammad Aghazadeh, Mehrdad Lotfi, Sahar Shakoei, Zahra Aghazadeh, Seyyed Mahdi Vahid Pakdel

**Affiliations:** a Dental and Periodontal Research Center, Department of Endodontics, Dental School, Tabriz University of Medical Sciences, Tabriz, IR Iran; b Department of Microbiology, Medicine School, Tabriz University of Medical Sciences, Tabriz, IR Iran; c Department of Oral Medicine, Dental School, Tabriz University of Medical Sciences, Tabriz, IR Iran; d Department of Prosthodontics, Dental School, Tabriz University of Medical Sciences, Tabriz, IR Iran

**Keywords:** Antibacterial Agents, Antifungal Agents, Mineral Trioxide Aggregate, Nanoparticles, Silver

## Abstract

**Introduction:**

Most current root-end filling materials do not provide a perfect seal. Thus, a microscopic space is likely to exist in the interface between walls of the root-end cavity and filling material, which allows microorganisms and their products to penetrate. In addition to good sealing ability and biocompatibility, root-end filling materials should ideally have some antimicrobial activity. Therefore, this in vitro study aimed to evaluate the antimicrobial properties of Angelus white mineral trioxide aggregate (MTA) and the mixture of MTA with silver nanoparticles (1% weight; MTA/SN).

**Materials and Methods:**

Antimicrobial properties of MTA and MTA/SN were tested by agar diffusion technique against Enterococcus faecalis, Pseudomonas aeruginosa, Staphylococcus aureus, and Candida albicans. The microbial inhibition zones around the materials were measured by a caliper with 0.1-mm accuracy. Student’s t-test was used for comparison between the two groups in normal data distribution and Man-Whitney U test for non-normal distribution.

**Results:**

Student’s t-test revealed that for E. faecalis, C. albicans, and P. aeruginosa, microbial inhibition zone of MTA/SN was significantly greater than that of MTA (P = 0.000). Mann-Whitney U test indicated no significant difference between the effect of MTA and MTA/SN on S. aureus (P > 0.05).

**Conclusion:**

Based on the results of this study, adding silver nanoparticles to MTA improved its antimicrobial efficacy.

## 1. Introduction

Ideal materials for sealing root-end cavities should prevent leakage, have dimensional stability, adhere to the cavity walls, resist resorption, and should be moisture resistant; they should also be nontoxic and biocompatible to promote healing. Because the majority of current root-end filling materials may not provide a hermetic seal, a microscopic space is likely to exist at the interface between root-end cavity and the filling material, along which bacteria and their products can penetrate. Thus, apart from other properties, root-end filling materials should ideally provide some antimicrobial activity (1-4).

Due to low solubility, low cytotoxicity, biocompatibility, and the ability to induce hard tissue formation, the mineral trioxide aggregate (MTA) has been used in many indications such as sealing the perforations, repair of external/internal root resorption, retrograde filling, pulp-capping agent in vital pulp therapy procedures, apexification, and recently, as intraorifice barrier; however, poor handling characteristics have been reported as one of the drawbacks of MTA (5-9).

Results of the studies conducted on antimicrobial properties of MTA are controversial. On the whole, it seems that MTA has limited antimicrobial properties. It was reported that the mixture of WMTA and 0.12% CHX exhibited higher antimicrobial efficacy (10, 11). However, it should be noted that adding CHX to WMTA can decrease its biocompatibility and compressive strength (10, 12, 13).

Silver nanoparticles (SN) are one of the most widely used nanoparticles, most notably serving as an antimicrobial agent for medical applications (14, 15). Small-sized SN can inhibit the growth of nitrifying bacteria more than that by

**Table 1.** Mean and standard deviation of growth inhibition diameters against tested microorganisms in millimeter

	MTA			MTA-SN		
**Microorganism**	Mean (SD)	95% CI for Mean		Mean (SD)	95% CI for Mean	
		Upper	Lower		Upper	Lower
**Enterococcus faecalis**	18.0685 (2.04989)	19.0279	17.1091	26.3615 (1.39201)	27.0129	25.7101
**Pseudomonas aeruginosa**	16.7100 (0.37683)	16.8864	16.5336	18.1102 (0.98889)	18.5628	17.6372
**Staphylococcus aureus**	15.8770 (0.5342)	16.1270	15.62698	16.0660 (0.58327)	16.3389	15.7930
**Candida albicans**	20.7857 (3.36106)	22.7263	18.8451	36.6744 (1.81304)	37.6405	35.7083

**Figure 1.**
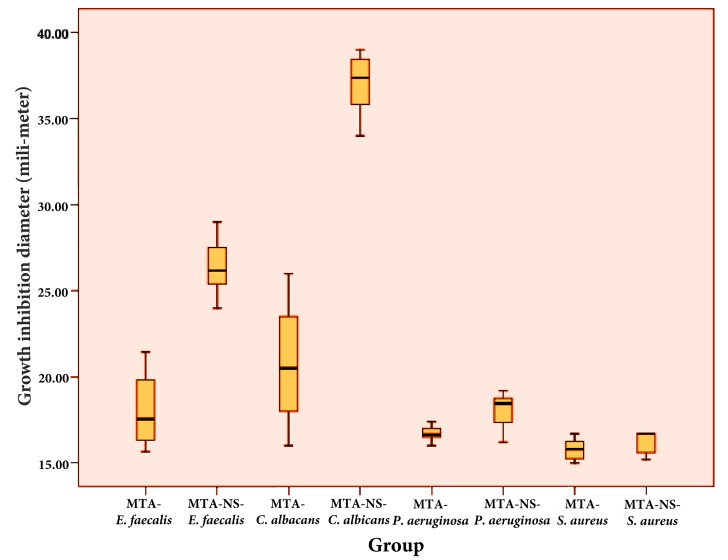
Box-plots of growth inhibition diameters against different bacteria (E. faecalis, C. albicans, P. aeruginosa, S. aureus)

silver ions at the same total silver concentrations (16, 17). The size of the particle was also related to antimicrobial activity; the smaller particles give more bactericidal effects compared to larger particles (18-20). Gomes-Filho et al. reported that SN dispersion was biocompatible, mainly at low concentrations (21). In an unpublished data by Lotfi et al., it is revealed that the biocompatibility of MTA and MTA/SN (1% weight) is similar in rat connective tissue.

It seems that silver nanoparticles have lower toxicity at low concentrations and have some antimicrobial effects. Therefore, the aim of this in vitro study was to evaluate antimicrobial properties of MTA and the MTA/SN mixture.

## 2. Material and Methods

White mineral trioxide aggregate (Angelus, Londrina, Brazil) with and without SN (Silver Nano-powder 7440-22-4, Sigma Aldrich, USA) by 1% weight was tested in this study. To prepare MTA/SN a digital weighing machine (AND GR-200 Analytical Balance, Lab Recyclers Inc., Gaithersburg MD, USA) was used in order to add SN by 1% weight to MTA.

Antimicrobial assessments were performed on three bacterial species, including Enterococcus (E.) faecalis (ATCC 29212), Pseudomonas (P.) aeruginosa (ATCC 15692), and Staphylococcus (S.) aureus (ATCC 29213), and the fungus Candida (C.) albicans (ATCC 10231). Agar diffusion method was used for the antimicrobial test. In this respect, double-layered approach was carried out. The base layer consisted of 10.0 mL of sterilized Muller-Hinton agar (MH; Difco, Detroit, MI, USA) poured into 20×100 mm sterilized Petri dishes. After solidification, a 5.0-mL seed layer, containing 106 colony-forming units/mL (0.5 in a McFarland nephelometer) was added to 5.0 mL of MH. All the inocula were taken from fresh cultures (18‒20-h culture). Three plates were prepared for each strain/material (i.e. a total of 24 plates). In each plate, 4 pits measuring 4 mm in depth and 6mm in diameter were prepared with sterile copper band and filled with separate materials (to avoid the interaction of different materials in a single plate), which were manipulated according to manufacturer’s recommendations. All the process was performed under a safety cabinet, and the control plates were used without adding any materials to indicate any other contamination during preparation process. The plates were maintained for 2 h at room temperature for pre-diffusion of the materials, and then incubated at 37° C for 24 h. The microbial inhibition zones around the materials were measured by a caliper with 0.1-mm accuracy. Data were analyzed by Kolmogorov-Smirnov normality test and if normal data distribution was obtained, Student’s t-test was used for comparison between groups and if not, Man-Whitney U test was applied. SPSS 16 software was employed for analysis.

## 3. Results

Means and standard deviations of growth inhibition diameters against different tested microorganisms are presented in Table 1. 

**Figure 2.**
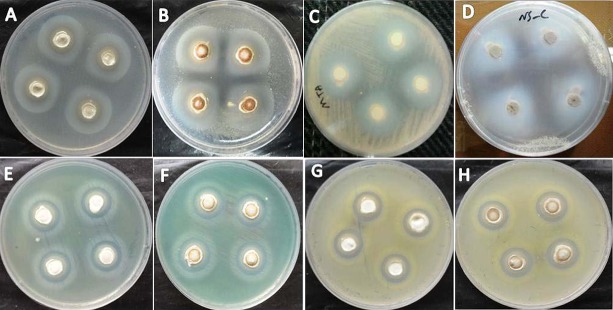
Microbial Inhibition zones; A. MTA against E. faecalis; B. MTA/SN against E. faecalis; C. MTA against C. albicans; D. MTA/SN against C. albicans; E. MTA against P. aeruginosa; F. MTA/SN against P. aeruginosa; G. MTA against S. aureus; H. MTA/SN against S. aureus.

Kolmogorov-Smirnov analysis revealed normal distribution of growth inhibition diameters in both MTA and MTA/SN groups against E. faecalis, C. albicans, and P. aeruginosa (P > 0.05). Student’s t-test revealed a significant difference between the effect of MTA and MTA/SN on E. faecalis, C. albicans, and P. aeruginosa (P = 0.000). Kolmogorov-Smirnov analysis showed non-normal distribution in both MTA and MTA/SN groups against S. aureus (P < 0.05). Mann-Whitney U test revealed no significant difference between the effect of MTA and MTA/SN on S. aureus (P = 0.415) (Figures 1 and 2). 

## 4. Discussion

Agar diffusion method was utilized in this study since it is one of the most commonly employed techniques for evaluating the antimicrobial activity of materials. The pre-diffusion period, which consists of maintaining the inoculated culture medium at room temperature for 2 h, is an important step in this method (22-26). This method has some limitations since it cannot distinguish between bacteriostatic and bactericidal effects (27); activities and the zones of inhibition are not only related to the inhibitory effects of the material, but also depend on the diffusibility of the material across the medium (28). Moreover, factors such as inoculum size, incubation time, and good material‒agar contact may also interfere with the results (25). However, if most of these variables are properly controlled, consistent and reproducible results may be obtained; subsequently, materials can be compared for their antibacterial effects under similar test conditions (25, 27).

The test bacteria selected were either true endodontic pathogens or associated with therapy-resistant cases (26-29). Although aerobic and facultative microorganisms are usually minor constituents of primary infections, they have been found with higher frequency in cases in which the treatment has been protracted, in flare-ups, and in failed cases (30). P. aeruginosa and E. faecalis are robust microorganisms which may infect the root canal (29, 31, 32) and E. faecalis is more likely to be found in cases of failed endodontic therapy than in primary infections (32, 33). On this basis, P. aeruginosa, and E. faecalis were used in this study. S. aureus is sometimes isolated from root canals and is known as a standard organism in antimicrobial testing (34). C. albicans is also predominant in persistent or refractory periapical lesions (29, 31, 35).

Gomes-Filho et al. evaluated the tissue response to implanted polyethylene tubes filled with fibrin sponge embedded with SN dispersion. They concluded that SN dispersion was biocompatible, mainly at low concentrations (21). Therefore, in this study low concentration (1% by weight) and small particles (< 150 nm) were used to reduce toxicity.

The results of this study revealed that adding SN by 1% weight to MTA improved its antimicrobial activity against E. faecalis, C. albicans, and P. aeruginosa. However, for S. aureus antimicrobial efficacy did not change. This antimicrobial efficacy enhancement was much higher for E. faecalis and C. albicans species but was less against P. aeruginosa, which might not lead to clinical improvement.

Antimicrobial activity of MTA seems to be associated with elevated pH values. Initial pH of MTA is 10.2, rising to 12.5 in 3h (2). It is known that pH levels of approximately 12 could halt the growth of most microorganisms, including E. faecalis (36). The antifungal effect of MTA might be attributed to its high pH or to substances that are released from MTA into the media (37).

Small-sized SN can inhibit the growth of nitrifying bacteria more than silver ions at the same total silver concentrations (16, 17). The size of the particle was also related to the antimicrobial activity; the smaller particles provide more bactericidal effects than larger ones [18-20].

Studies have also focused on the potential antimicrobial activity of SN (18-20, 38). Baker et al. (19) found that silver concentration as low as 8 mg/cm2 had a cytotoxic effect on Escherichia (E.) coli. They also showed significant in vitro antimicrobial activity and prevention of biofilm formation by using E. coli, Enterococcus, S. aureus, Staphylococci, P. aeruginosa, and C. albicans by coating catheters (39).

Compared to similar studies (11, 37, 40-42), MTA exhibited good antifungal activity against C. albicans in this study. Addition of SN to MTA, improved its antifungal efficacy. Similarly, in an in vitro study, silver-zeolite was incorporated into MTA, which resulted in better antifungal activity compared to pure MTA (42).

Although silver is known to possess antibacterial properties, its exact mechanism of action is not fully understood. Three possible theories could be considered: i) silver ions destroy the cell wall; ii) silver ions interrupts the RNA replication sequence of the microorganism, thereby prevents cell multiplication; and iii) by blocking cellular respiration, silver ions effectively suffocates the bacteria (43). Another possible antibacterial mechanism of silver ions is the interaction with thiol groups in proteins, inducing the inactivation of bacterial proteins (44).

Some investigations replaced distilled water with other liquids to mix with MTA powder in order to enhance its antimicrobial activity (11, 12). Based on the reported results, it appears that enhancing antibacterial property of MTA by adding various liquids might adversely affect other properties of the material (45). In this study, antimicrobial activity of MTA improved with incorporation of silver nanoparticles. According to Lotfi et al., addition of 1% of SN to MTA did not alter its biocompatibility contrary to other studies in which adding CHX to MTA diversely affected its biocompatibility (11, 12). However, further studies are necessary to evaluate the properties of this mixture. 

Since some cytotoxic effects have been observed in SN, it might be logical to investigate the antimicrobial effect of some nanoparticles other than silver to improve the antimicrobial activity of MTA. Regarding the limitations of disc diffusion test, other methods such as liquid dilution could be used in further studies.

## 5. Conclusion

Based on the findings of this in vitro study, adding silver nanoparticles to Angelus white MTA enhanced its antimicrobial activity against E. faecalis, C. albicans, and P. aeruginosa.
